# Species-specific allometric models for reducing uncertainty in estimating above ground biomass at Moist Evergreen Afromontane Forest of Ethiopia

**DOI:** 10.1038/s41598-023-51002-6

**Published:** 2024-01-11

**Authors:** Abu Mulatu, Mesele Negash, Zerihun Asrat

**Affiliations:** 1 Ethiopia Forest Development, Dire Dawa Center, P.O. Box 1708, Dire Dawa, Ethiopia; 2 Hawassa University, Wondo Genet College of Forestry and Natural Resources, P.O. Box 128, Shashemene, Ethiopia

**Keywords:** Forest ecology, Forestry

## Abstract

An allometric equation is used to convert easily measured tree variables into biomass. However, limited species-specific biomass equations are available for native tree species grown in various biomes of Ethiopia. The available pantropic generic equation has resulted in biases owing to the uncertainty of the generic model estimation due to the difference in tree nature and response to growth conditions. The objective of the study is, thus, to develop a species-specific allometric equation for reducing uncertainty in biomass estimation at the Moist Evergreen Afromontane Forest in south-central Ethiopia. Five tree species were selected for model development, these selected trees were harvested and weighed in the field. The measured above-ground biomass data related to easily measured tree variables: diameter at stump height, diameter at breast height (dbh), crown diameter, and total tree height. The developed model evaluated and compared with previously published model by using measures of goodness of fit such as coefficient of determination (R^2^), total relative error, mean prediction error, root mean square error, and Akaike information criteria. The analysis showed that a model with dbh as a single predictor variable was selected as the best model for the estimation of above-ground biomass. It gives the highest R^2^ for Syzygium guineense (0.992) and the lowest for Bersama abyssinica (0.879). The additions of other tree variables did not improve the model The pantropic model by Brown overestimates the biomass by 9.6–77.8% while both Chave models resulted in an estimation error of 12–50.3%. Our findings indicated that species-specific allometric equations outperformed both site-specific and pantropic models in estimating above-ground biomass by giving 0.1% up to 7.9% estimation error for the respective tree species.

## Introduction

Moist Evergreen Afromontane Forest is one of the vegetation types in Ethiopia and it is located in the western highland, the patch of Wondo genet, and the southern slope of Bale Mountain^1^. The Moist Evergreen Afromontane Forest, as cited by Gole et al.^2^, serves as a habitat for wild coffee. Moreover, it plays a pivotal role in the conservation of highland forest birds and plant diversity. It provides various benefits for the local people like coffee production, honey, spices, fuel wood, construction wood, farm tools, and grazing land. Additionally, it has a role in climate change mitigation by sequestering and storing carbon. Despite this, it faces threats of deforestation and degradation^3^ from agricultural expansion, illegal cutting, and overgrazing.

In Ethiopia, there is a high rate of Forest degradation and deforestation. from 2000 to 2013 the country lost a mean of 92,000 ha forest annually^4^, and the majority of this share was taken by Moist Evergreen Forest next to *Combretum-Terminalia* woodlands. To address the problem, Ethiopia engaged in international initiatives like REDD + (reduction of deforestation and degradation; conservation and sustainable forest management; and enhancing forest carbon). Hence, measuring and determining the biomass and forest carbon stock is the center of attention. Besides, understanding the carbon balance of forest ecosystems is vital to minimize the impact of climate change, and exploring mitigation options^5^. The biomass of the individual tree is determined in two ways. The first method involves cutting down the tree, weighing it all, and converting it into dry biomass. The second method involves converting measured tree variables (Diameter, Height, and Crown) into biomass using an allometric equation. The second approach is feasible and environmentally sound.

An allometric equation is a method that establishes a relation between some easily measured tree dimensions (height, diameter, and crown diameter) and the dependent variable, total above-ground biomass. It gives an insight into the carbon sequestration potential of woody vegetation. As well, it also helps to calculate the costs and benefits associated with forest carbon projects and improves bio-energy systems and sustainable forest management^6^. It can be a generic, site-specific, or species-specific allometric equation. Some scholars^7–10^ are in favor of using species-specific allometric equations. Additionally, studies indicate that the application of a generic equation leads to uncertainty in the estimation of biomass^11–15^. Species-specific allometric equations can reflect biomass variance caused by differences in tree nature (number of stems, height to branches), age, diameter, stand density, cultivars, site environment (climate and soil), and management approach.

In Ethiopia there is growing interest in developing allometric equations, for example, some researchers develop site-specific allometric equations^11–15^ and species-specific^12,16^. In Moist Evergreen Afromontane Forest, only a limited number of tree species developed species-specific allometrics. For example, *Olea europaea* by Kebede and Soromessa^17^; *Diospyros abyssinica* by Daba and Soromessa^18^; *Albizia grandibracteata* and *Trichilia dregeana* by Daba and Soromessa^19^. Even though the country has made significant efforts to develop allometric equations at site and species level. According to Sebrala et al.^20^, Ethiopia lacks sufficient national models for assessing forest biomass and carbon stocks and monitoring changes using Tier-2 and Tier-3 methodologies recommended by the IPCC. Additionally, there are issues with the developed model's inclusion of wood basic density as a predictor variable in species-specific allometric equations, taxonomic naming issues, and the notion that models are best fitted when they have negative parameter estimators. So, the present study aims to develop allometric equations for estimating above-ground biomass for five selected tree species in the Moist Evergreen Afromontane Forest of South-Central Ethiopia. The specific objectives were to develop species-specific allometric equations for the estimation of above-ground biomass for five native tree species; to develop a biomass model for the tree compartment of this selected tree; to evaluate and compare the newly developed allometric equations with the existing site-specific and generic allometric equations.

## Materials and methods

### Study site location

**Figure 1:**
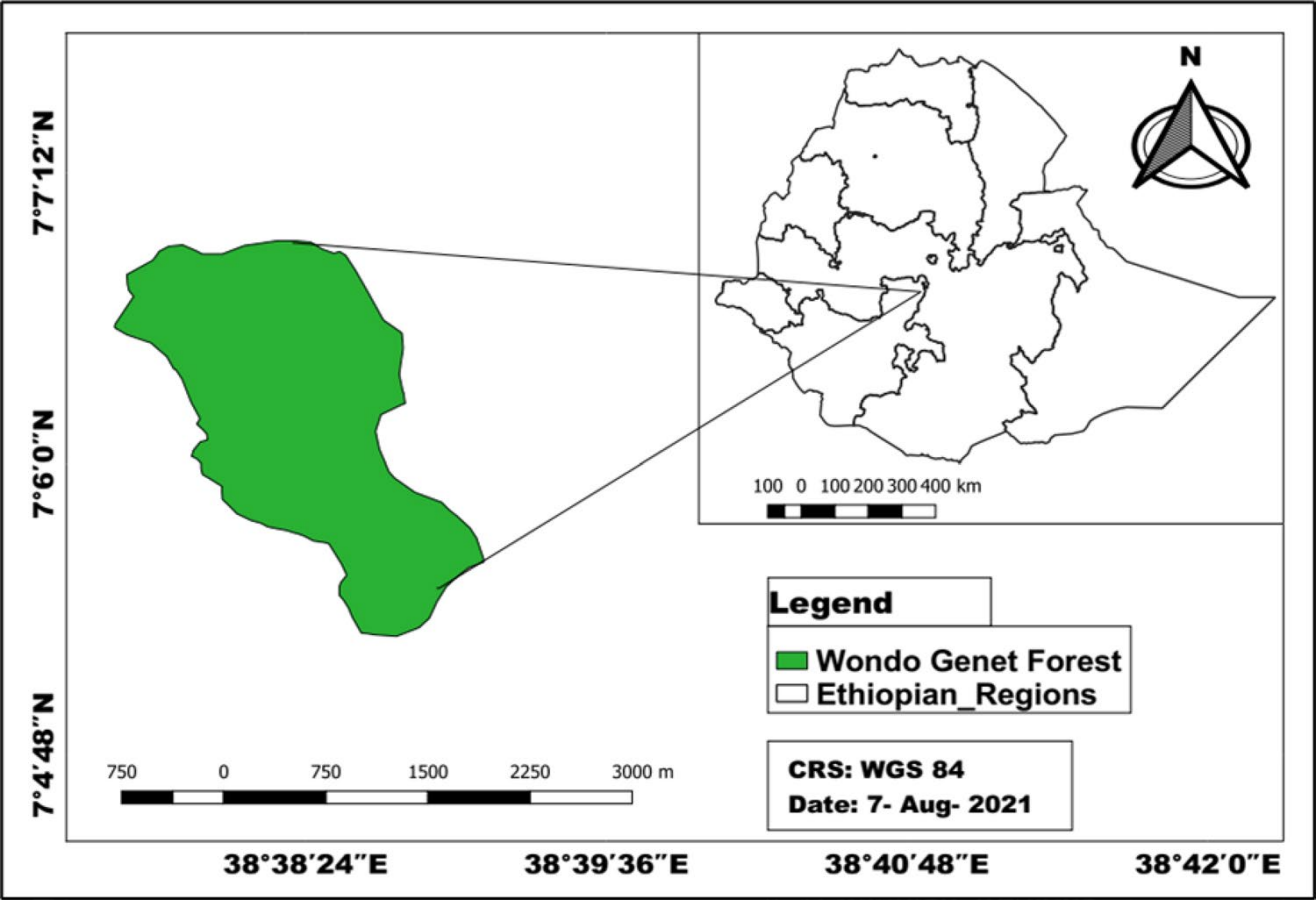
Map of the study area.

The study site (or area) was located in the Wondo Genet natural forest on the southeast part of Ethiopia (Fig. 1), [N 7° 5.4′–N 7° 7.2′, and E 38° 38.4′–E 38° 40.4′]. The altitude ranges from 1850 to 2400 m.a.s.l. It is categorized in the remnant Moist Evergreen Afromontane Forest^21^ located in the protected and inaccessible mountain chains of Abaro. The area has a mean annual rainfall and temperature of 1200 mm and 22 °C respectively^22^ with bimodal rainfall distribution with longer precipitation from June to October and lower from March to April^23^. The topography of the study area has 43.5% mountains and hills, 36.25% flat areas, and 20.25% undulating parts of the district^24^. The soils are young and of volcanic origin, characterized by well-drained loam or sandy loam. The soil pH of the study area is between 5.6 and 6.5^25^.

### Data collection

#### Sampling design and sample tree selection

To estimate the number of native trees to be included in the development of the allometric equation and to observe how tree species are distributed over the diameter range, tree inventory data collected by Asrat et al.^26^ were used. Based on this inventory result, the dominance of tree species was calculated based on the following formula.

1 $${\text{Basel}}\;{\text{area}} = \left( {\pi d^{2} } \right)/4$$

where “d’” is the diameter of the tree. Accordingly, five tree species (i.e.: *Albizia gummifera, Bersama abyssinica, Croton macrostachyus, Vepris dainellii, and Syzygium guineense)* were selected for the development of the allometric equations. A total of 59 trees were harvested with a minimum diameter of 5 cm and a maximum diameter of 106.5 cm for the development of the allometric equation and covers 32.0% of the basal area. . The diameter classes were formulated with a 10 cm diameter interval for each tree species. Representative sample trees were distributed in diameter class based on the basal area proportion and sample trees were selected systematically within the diameter class. Trees having unusual forms such as broken crowns and stem knots were removed from the selection in model development unless they represent a significant portion of the forest, and trees grown in the unrepresentative site such as forest edge were not included. Hence, trees that are free from broken branches and defects were selected for harvesting^27^. Before the tree felled, each sample tree from each species was identified and located with a GPS coordinate point and marked by the researcher and one local guide. Number and statistical summary of sampled trees; and distribution of the harvested tree with diameter class for each tree species presented in (Table 1).

**Table 1:** Summary (Range, Mean, and Standard error) of biometric attributes of the harvested sample trees.

Tree species	N	dbh(cm)	dsh(cm)	Ht(m)	Cd (m)	AGB (kg)
*A. gummifera*	13	5.8–106.5 (44.2)(8.4	8.6–117.5 (50)(9.4)	6.0–38.8 (20.9)(2.4)	2.0–25.0 (13.6)(2)	8.83–12,433.4 (2705)(1060.9)
*C. macrostachyus*	14	5.0–81.0 (38.6)(5.9)	8.0–98.0 (45)(6.9)	6.5–27.8 (21)(1.9)	2.0–25.0 (12)(2)	5.9–5403.1 (1175)(395.5)
*V. dainellii*	11	5.0–41.6 (22.1)(3.4)	6.0–61.3 (27)(4.6)	4.0–17.3 (11.7)(1.3)	2.0–19.0 (12.7)	12.3–1130.3 (389)(226.7)
*S. guineense*	11	6.0–95.0 (41.9)(9.3)	10.0–110 (52)(10.7)	8.0–25.0 (16.9)(1.7)	3.0–26.0 (12.5)(2.7)	13.9–5738-5 (1551)(587.6)
*B. abyssinica*	10	6.0–44.0 (23.1)	8.0–51.0 (26)	6.0–16.3 (12.2)(1.3)	1.5–13.0 (7.2)(1.7)	12.3–1130.3 (389)(116.9)

### Biomass determination

The destructive method was employed for the determination of the biomass of individual trees. After the tree diameter at stump height (0.3 m), diameter at breast height (1.3 m) (if buttress occurred tree diameter measured above buttress 0.3), and crown diameter was recorded. The tree was cut down closest to the ground and the total tree height using a tape meter was measured. The felled tree is sorted into three main sections: stem (stump plus to top > 10 cm diameter), branches (tree parts apart from the main stem and diameter > 2 cm), and foliage (leaves, twigs, small branches diameter < 2 cm, and fruit part). The section of all felled trees weighed independently in the field using a hanging balance (200 kg capacity). The weight of the stump was determined by using the volume of the stump and the wood's basic density.

For the determination of foliage dry to fresh ratio, the foliage 200-250 g sample was taken from each tree. Additionally, four disks constitute three from the stem part, and one from the branch to determine the dry-to-fresh weight ratio of stem and branch. The fresh weight of the sample was measured immediately in the field to avoid moisture loss. Then after labeling, the sample was transported to the WGCF-NR laboratory for oven-drying at 72 C for foliage, and 103 C for the wood part until it reached a constant weight^27^. The dry weight of the sample was determined by digital balance (± 0.1 g). Finally, the dry weight of each section was determined by taking the dry-to-fresh weight biomass ratio.

### Data analysis

**Figure 2:**
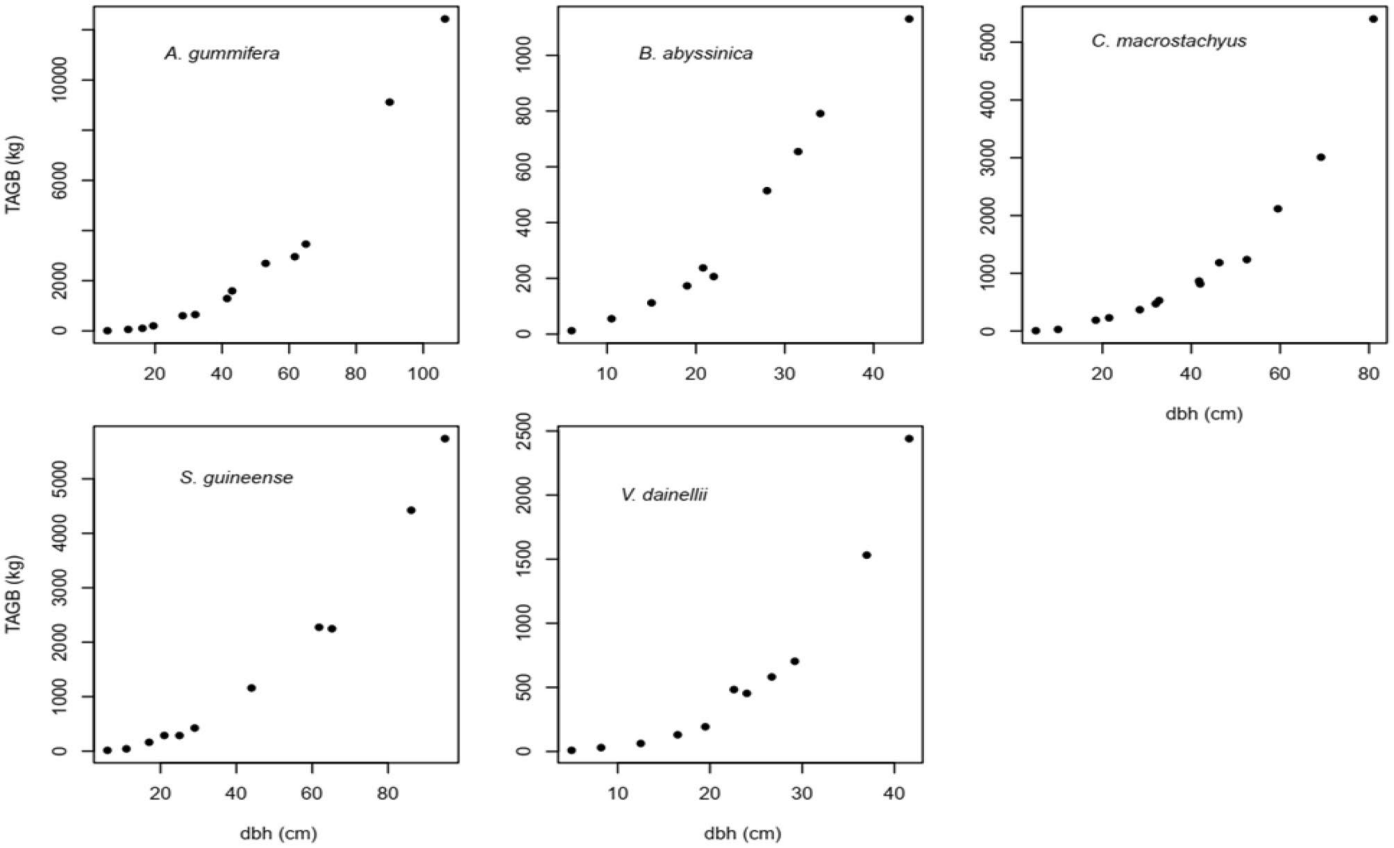
Scatter plot dbh (cm) against Total above-ground biomass (TAGB) (kg).

The data analysis was undertaken in R software version 4.01^28^ by using 'nlstools' package^29^. Prior to conducting the analysis, an investigation was performed by plotting a scatter plot (Fig. 2) to examine the relationship between dependent variable (above ground biomass) and independent variable (diameter at breast height). Accordingly, nonlinear relationship between the independent and dependent variables were observed. As a result, nonlinear regression methods were established on power model. Consequently, power models used in several studies^14,15,26^ were tested in the present study. Based on this we formulated six different model forms for testing the Species-specific biomass models, by using dbh and dsh as sole predictors and combined with a stepwise inclusion of ht and crw.

Additionally, weighted regression was employed to reduce the heteroscedasticity in nonlinear regression^30^. It is the method of data transformation used in our data set to remove the error variance. Based on the procedure adopted by Picard et al.^27^, the weighting Factor (“c”) will be developed for each tree species. Finally, the weight will be = $\frac{1}{{({\text{dbh}})}^{{\text{c}}}}$ ; where dbh, is the diameter of the tree, and “c” is the weight factor.

$${\text{AGB }} = a{* }\left( {dbh} \right)^{b} \quad \left( {{\text{Model 1}},{\text{ M1}}} \right)$$

$${\text{AGB }} = a{* }\left( {dbh} \right)^{b} {*}\left( {ht} \right)^{c} \quad \left( {{\text{Model 2}},{\text{ M2}}} \right)$$

$${\text{AGB }} = a{* }\left( {dsh} \right)^{b} \quad \left( {{\text{Model 3}},{\text{ M3}}} \right)$$

$${\text{AGB }} = a{* }\left( {dsh} \right)^{b} {*}\left( {ht} \right)^{c} \quad \left( {{\text{Model 4}},{\text{ M4}}} \right)$$

$${\text{AGB}} = {\text{a}}* \left( {{\text{dbh}}} \right)^{{\text{b}}} *\left( {{\text{cd}}} \right)^{{\text{c}}} \quad \left( {{\text{Model}}\;5,{\text{M}}5} \right)$$

$${\text{AGB }} = a{* }\left( {dbh} \right)^{b} {*}\left( {ht} \right)^{c} {* }\left( {cw} \right)^{d} \quad \left( {{\text{Model 6}},{\text{ M6}}} \right)$$

where: AGB is the biomass of the tree in (kg), dbh is the tree diameter at breast height (cm), ht is tree height in (m), dsh is the diameter at stump height (cm), cd is the crown diameter in (m) and a, b, c, d, are model parameters.

### Model evaluation and comparison

For evaluating the models' performance, cross-validation specifically leave one out cross-validation (LOOCV, where a model fitted to the ‘n-1’ dataset and then performance is assessed on the single observation left out and repeats the procedure n times until all observations covered by the process) was used. It is an efficient method of model validation, where every data set is used for training and test data^31^. This kind of model validation is important when a small data set exists^36^. Furthermore, it has no randomness since each observation is used as a training and validation. Then the developed allometric models were evaluated through goodness-of-fit measures such as mean prediction error (MPE), root mean square error (RMSE), Akiaka information criterion (AIC), True Relative Error (TRE), and R-square. Thus, models that recorded the lowest value of MPE, RMSE, AIC, and the higher values − R^2^ were selected. Paired t-tests were used to see the difference between observed and predicted values. Pearson’s correlation test was also used for testing the correlation between AGB and independent variables (dbh, dsh, crown diameter, and height).

2 $$\mathrm{RMSE }=\sqrt{\frac{1}{n}(\sum_{i=1}^{n}(yi-}{\widehat{ yi})}^{2}$$

3 $${\text{RMSE}}\% = \frac{RMSE}{{\overline{y}}}* 100$$

4 $${\text{MPE}} = \frac{1}{n}{ }\mathop \sum \limits_{i = 1}^{n} (yi - \widehat{{{ }yi}})$$

5 $$\mathrm{MPE\% }=\frac{{\text{MPE}}}{\overline{{\text{y}}} }* 100$$

6 $$\mathrm{TRE\% }=\frac{\sum \widehat{ {\varvec{y}}}i-yi}{\sum yi}*100$$

7 $$R^{2} = 1 - \frac{{\mathop \sum \nolimits_{i = 1}^{n} (y_{i} - \overbrace {{y}_{i}} )^{2} }}{{\mathop \sum \nolimits_{i = 1}^{n} (y_{i} - \overline{y})^{2} }}$$

where: MPE is the mean prediction error, RMSE is a root mean square error, ${\varvec{y}}i$ is the observed value of the ith sample tree, $\widehat{{\varvec{y}}}i$ is the predicted value of the ith sample tree, $\overline{\mathbf{y} }$ is the mean observed value and n is the number of observations.

To compare model performance, the best-ranked model in the present study was used to compare it with previously developed pantropic^32–34^ and site-specific models^26^. The models used in the comparison are presented in Table 2.

**Table 2:** Selected previously published model for comparison.

Reference	Model
Brown^36^	ABG (kg) = 42.69–12.800*(dbh) + 1.242*(dbh)^2
Chave, et al.^34^	AGB (kg) = 0.0509*wbd*dbh^2*ht
Chave, et al.^33^	AGB (kg) = 0.0673*(wbd*dbh^2*ht) ^0.976
Asrat et al.^26^	AGB = 0.21765 × (dbh)^1.77660 × (ht)^0.33242 × (crw)^0.65575 × (wbd)^1.07739

### Ethical approval and consent to participate

The collection of plant material and the performance of experimental research on such plants complied with the national guidelines of Ethiopia.

## Results

### Correlation between tree variables and different biomass components of the tree

Spearman correlation between the independent and dependent tree variables is presented (Table 3). For all tree species tree dependent variable, has a strong relationship with dbh. For the *C. macrostachyus* tree, height has a weak relationship with the tree-dependent variable. Regarding crown diameter, *B. abyssinica* doesn’t strongly correlate with all tree’s dependent variables except merchantable stem biomass. One of the factors for the variation of the correlation between tree species and different biomass compartments nature of species and growth conditions. Based on the nature of the tree species and growing conditions the relation between the tree variable and the biomass component of the tree will be affected^35^.

**Table 3:** Correlation Matrix between dependent and independent tree variables.

Species	Compartment	Tree variable			
		Dsh	dbh	ht	cd
*A. gummifera*	Foliage biomass	0.97***	0.97***	0.87***	0.87***
	Branch biomass	0.88***	0.91***	0.75***	0.70**
	Merchantable Stem biomass	0.93***	0.95***	0.8***	0.76***
	Total Above ground biomass	0.91***	0.94***	0.78***	0.74***
*C. macrostachyus*	Foliage biomass	0.91***	0.93***	0.72*	0.59
	Branch biomass	0.95***	0.94***	0.73*	0.43
	Merchantable Stem biomass	0.98***	0.97***	0.83**	0.45
	Total Above ground biomass	0.98***	0.97***	0.79**	0.45
*S. guineense*	Foliage biomass	0.93***	0.92***	0.63	0.93***
	Branch biomass	0.90***	0.88***	0.54	0.85***
	Merchantable Stem biomass	0.90***	0.88***	0.60	0.86***
	Total Above ground biomass	0.91***	0.90***	0.59	0.87***
*V.dainellii*	Foliage biomass	0.94***	0.95***	0.86***	0.91***
	Branch biomass	0.91***	0.94***	0.81**	0.89***
	Merchantable Stem biomass	0.95***	0.98***	0.87***	0.92***
	Total Above ground biomass	0.94***	0.96***	0.85**	0.91***
*B. abyssinica*	Foliage biomass	0.94***	0.89***	0.76**	0.69
	Branch biomass	0.93***	0.85***	0.72	0.64
	Merchantable Stem biomass	0.91***	0.94***	0.87	0.76*
	Total Above ground biomass	0.94***	0.90***	0.79**	0.70

dsh, diameter at stump height; dbh, diameter at breast height; ht, total tree height; and cd; crown diameter; Significance level: *p < 0.05; **p < 0.01; ***p < 0.001.

### Species-specific allometric equation for the selected tree species

The best-performed allometric equation with a measure of goodness-of-fit for the five tree species for all compartments is presented (Table 4) and all tested models are presented (Appendix A and B) The selected model holds the highest R-square, lowest RMSE, MPE, and AIC. The model that gives negative and insignificant parameters is not considered a valid model. Accordingly, for the total above-ground biomass (TAGB) model (M1), with dbh sole predictor gives significant parameter estimates for all tree species. And explained 99.3% of biomass variation for *S. guineense* whereas the lowest by *B. abyssinica* 89.3%. Whereas M3 with dsh as a single predictor variable explained 97.8% of the variation in the case of *A. gummifera* followed by *C. macrostachyus* (97%), and *V. dainellii* (72%)*.* The addition of other tree variables in the model didn’t improve the model performance and resulted in a negative regression coefficient in some cases.

**Table 4:** Selected species-specific biomass model for all tree compartments and model performance evaluation.

Species	Tree compartment	Model	Parameter estimates			Adj. R	RMSE		MPE		AIC
			a	b	c		Kg	%	Kg	%	
*A. gummifera*	Foliage	M3	0.08*	1.87**		0.85	57.29	36.73	− 8.63	− 5.53	118
	Branch	M2	0.013*	3.21***	0.54*	0.92	454.14	40.23	− 14.46	− 1.28	141
	M. stem	M1	0.15**	2.29***		0.97	273.12	19.23	− 49.4	− 3.48	158
		M3	0.099*	2.32***		0.94	428.08	30.14	− 130	− 9.19	159
	TAGB	M1	0.145 **	2.440***		0.99	384.194	14.202	− 26.43	− 0.97	161.26
		M3	0.086*	2.49 ***		0.98	547.76	20.25	50.34	1.86	169.13
*S. guineense*	Foliage	M1	0.202*	1.62***		0.88	42.54	35.50	13.65	11.39	102
		M3	0.05**	1.87***		0.90	37.89	31.62	7.73	6.45	91
	Branch	M1	0.02***	2.53***		0.98	101.0	15.85	17.60	2.76	96
	M. stem	M1	0.24*	2.07***		0.93	213.14	26.86	− 76.19	− 9.60	165
		M4	0.14*	2.72***	0.74*	0.97	145.59	18.35	− 31.29	− 3.94	125
	TAGB	M1	0.287***	2.18***		0.992	158.129	10.198	− 62.702	− 4.04	125.50
*V. dainellii*	Branch	M1	0.012*	3.09***		0.89	143.36	43.84	19.20	5.87	103
		M3	0.006**	3.13***		0.51	298.45	91.26	− 55.28	− 16.9	100
	M. stem	M1	0.063**	2.50***		0.83	81.44	39.20	− 6.33	− 3.04	77
	TAGB	M1	0.099**	2.65***		0.918	198.517	30.604	52.423	8.082	109.85
		M3	0.073**	2.61 ***		0.69	389.02	59.97	− 0.70	− 0.11	119.73
*B. abyssinica*	M. stem	M2	0.05*	1.67***	1.14**	0.98	21.73	10.49	− 0.61	− 0.29	85
	TAGB	M1	0.254*	2.25***		0.879	114.95	29.58	− 17.04	− 4.38	98.46
*C. macrostachyus*	TAGB	M1	0.085*	2.49***		0.918	392.90	33.445	43.721	3.722	170.11
		M3	0.085*	2.39***		0.97	245.29	20.88	31.18	2.65	172.82

M.stem, Merchantable stem Diametr > 10 cm; TAGB, Total above-ground biomass; Significance level: *p < 0.05; **p < 0.01; ***p < 0.001; RMSE, root mean square error, AIC, Akaike information criterion; Adj. R, adjusted R square; MPE, mean prediction error.

Based on the analyzed result, M3overestimated the foliage biomass by 8.6 kg for *A. gummifera,* while M1 underestimated by 13.7 kg for *S. guineense*. For *B. abyssinica* including height with dbh results in better estimation by explaining 98.6% of the variation in Merchantable stem biomass. Correspondingly, M4 with dsh and height results in a better estimation of Merchantable stem biomass, by overestimating the biomass with 31.3 kg for *S. guineense.* In *C. macrostachyus,* all tested models for the tree like Foliage, Branch, and Merchantable stem resulted in insignificant regression coefficients.

**Figure 3:**
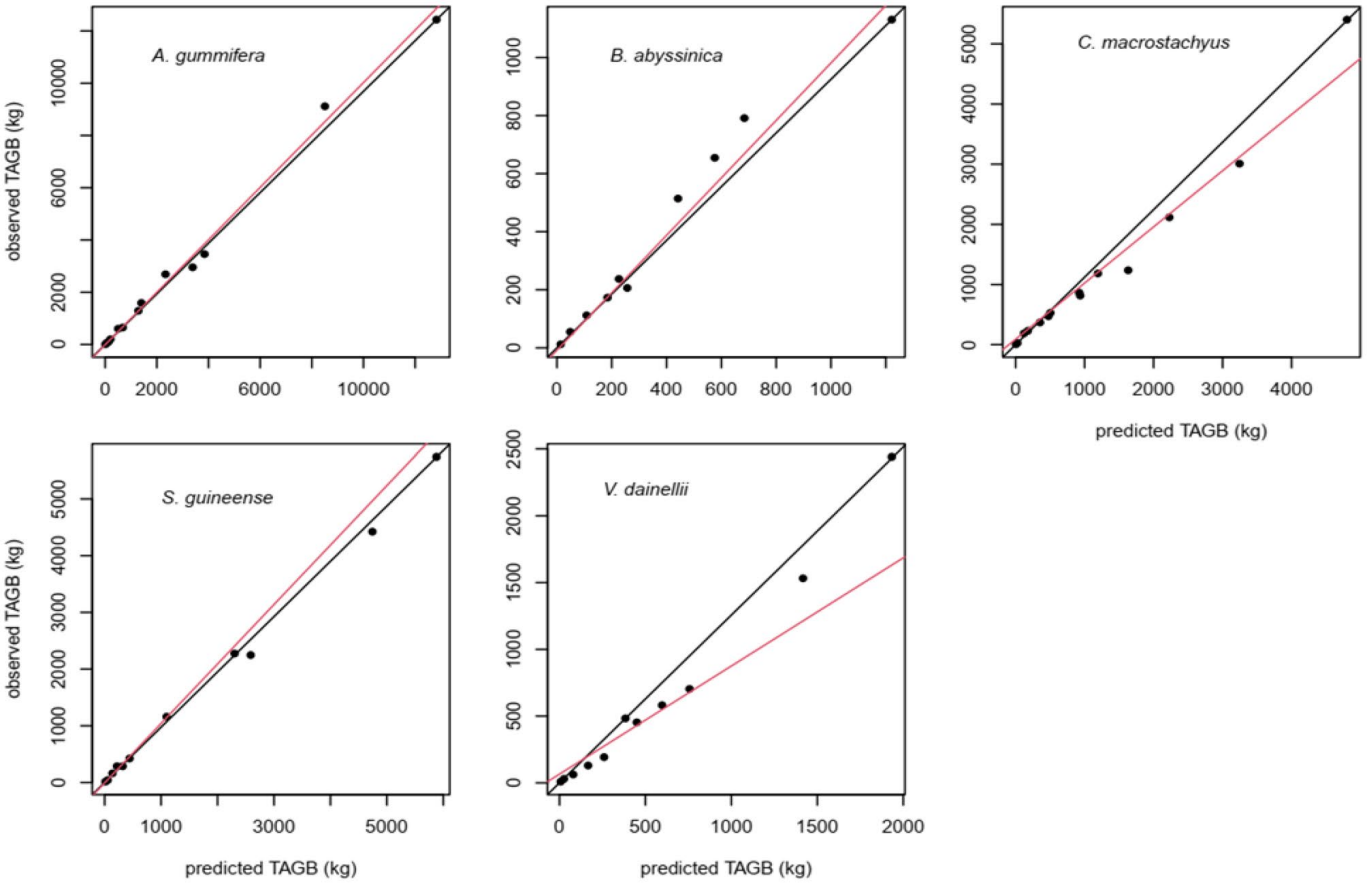
The relationship between the observed and predicted total ABG of the five tree species. The red line represents the line that best fits the residuals, while the black line represents the 1:1 line.

The observed and predicted AGB for the selected model were plotted in (Fig. 3). The result showed that there is no significant difference between the observed AGB and predicted AGB for the best-selected model. Based on the P-value there is no proof to reject the null hypothesis (intercept = 0 and slope = 1). However, the discrepancy between observed and predicted varies between tree species.

### Biomass model comparison with the previous study

The comparisons were made by applying the previously published generic allometric model, site-specific model, and selected model M1 of the current study on our data, and the result is shown in (Table 5). The model by Brown^36^ overestimates the AGB For *S. guineense* (77.8%)*, C. macrostachyus* (66.4%)*, B. abyssinica* (43.2%), and *A. gummifera* (9.6%). On the other hand, a model developed by Chave et al.^33,34^ underestimate the AGB for *A. gummifera* (17.8% and 17.4%), *V. dainellii* (50.3% and 47.4%), and *B. abyssinica* (21.2% and 16.4%), respectively, whereas overestimate the AGB for *S. guineense* (15.8% and 17.1%) and overestimate for *C. macrostachyus* (9.7% and 12.4%), respectively. The site-specific model developed by Asrat et al.^26^ overestimates the biomass for *C. macrostachyus* (37.4%) and *S. guineense* (71.9%). In all tree species, the currently developed model M1 has the least prediction error with the highest for *V. dainellii* (7.3%) whereas the lowest is *B. abyssinica* (0.1%). The comparison based on the value of Total relative Error (TRE) and Mean prediction Error (MPE) indicates that for all tree species, the developed model in the current study outperformed the previously developed model.

**Table 5:** Comparison of the selected general models and previously published both generic and for each species.

Species	Previously developed models	predicted AGB mean in (Kg)	SE	RMSE		MPE		TRE (%)
				Kg	%	Kg	%	
*A. gummifera*	Chave et al.^33^	2233.5	900.74	951	35.2	471.8*	17.4	− 17.4
	Asrat et al.^26^	2576.9	887.43	567.2	21	128.3	4.7	− 4.7
	Chave et al.^34^	2223.2	949.08	936.7	34.6	482.0*	17.8	− 17.8
	Brown^36^	2964	1074.33	434.9	16.1	− 258.8*	− 9.6	9.6
	M 1	2716.6	1067.48	284.4	10.5	− 11.4	− 0.4	0.04
*C. macrostachyus*	Chave et al.^33^	1320	377.97	318.6	27.1	− 145.2*	− 12.4	12.4
	Asrat et al.^26^	1614.2	379.83	697.1	59.3	− 439.4**	− 37.4	37.4
	Chave et al.^34^	1289.1	521.24	296.1	25.2	− 114.3	− 9.7	9.7
	Brown^36^	1955.4	562.35	1038.5	88.4	− 780.6***	− 66.4	66.5
	M 1	1190.4	372.84	207.8	17.7	− 15.7	− 1.3	1.07
*S. guineense*	Chave et al.^33^	1815.4	703.59	483.1	31.2	− 264.8*	− 17.1	17.1
	Asrat et al.^26^	2664.9	701.36	1913.4	123.4	− 1114.3*	− 71.9	71.9
	Chave et al.^34^	1795.7	1067.79	479.2	30.9	− 245.1*	− 15.8	15.8
	Brown^36^	2757.6	1061.76	1930.2	124.5	− 1207.0*	− 77.8	77.8
	M 1	1601.1	616.78	130.8	8.4	− 50.5	− 3.3	2.5
*V. dainellii*	Chave et al.^33^	341.2	98.37	16.8	79.7	307.5*	47.4	− 46.9
	Asrat et al.^26^	632.8	102.51	219.6	33.9	15.9	2.5	1.0
	Chave et al.^34^	322.1	179.04	322.1	82.9	326.6*	50.3	− 49.8
	Brown^36^	545.5	160.18	263.4	40.6	103.2	15.9	− 15.0
	M1	601	185.09	158.5	24.4	47.7	7.3	− 8.2
*B. abyssinica*	Chave et al.^33^	324.9	102.42	89.3	23	63.8**	16.4	− 16.4
	Asrat et al.^26^	393.2	106.67	64.1	16.5	− 4.5	− 1.2	1.2
	Chave et al.^34^	306.2	113.55	108.3	27.9	82.4**	21.2	− 21.2
	Brown^36^	556.7	183.85	271.2	69.8	− 168.0*	− 43.2	43.2
	M1	389.2	117.25	59.4	15.3	− 0.6	− 0.1	0.02

M1, The best selected model in the present study; SE, Standard Error; Significance level: *p < 0.05; **p < 0.01; ***p < 0.001.

## Discussion

### Species-specific allometric equation for the selected tree species

A considerable proportion of variations in the total above-ground biomass (TAGB) are explained by the dbh as a sole predictor variable in each tree species case. The highest explaining potential of dbh is present in *S. guineense* (99.3%), and the lowest in *B. abyssinica* (89.3%), This finding is similar to those reported in several studies^12,17,37,38^.

The addition of tree height in the model results in a negative regression coefficient and doesn’t improve model performance. This finding is inconsistent with some studies^39–41^. Tree allometry is affected by differences in tree nature (number of stems, height to branches), age, diameter, stand density, cultivars, site condition (climate and soil), and management practice^42^. For example, a tree that grows in an open forest will have a shorter height than a tree that grows in a closed forest for the given diameter. This also affects the relationship the biomass and tree height^43,44^. Due to this diameter is the most important tree variable in the estimation of biomass.

Regarding crown diameter, the inclusion of this predictor variable in the model did not improve the biomass estimation. This finding is inconsistent with some studies^14,15,26^. The forest ecosystem of Wondo Genet was exposed to disturbance from fuelwood collectors, illegal logging, and man-made fire^21,23^. This reduces competition for the upper canopy and in this type of forest, trees invest more in diameter than height^45^ and tree allometry will be changed. In this case, dbh will be an important predictor for the estimation of above-ground biomass. Besides, dsh is another important tree variable explaining the variation existing in biomass and performed better than dbh for tree species like *C. macrostachyus*, but because of the measurement difficulty in natural forests, models that include dsh are not recommended as the best option for further application.

### Species-specific biomass model comparisons with previous study

The best-ranked selected model was compared with one site-specific developed generic allometric equation and three pantropic allometric equations. Additionally, different statistics are used as performance indicators to evaluate the performance of each model. A pantropic model developed by Brown^36^, overestimates the biomass by 9.6–77.8%. As well, Chave et al.^33^ gave a prediction error range of 12.4–47.4%; and Chave et al.^34^ resulted in an error range from 15.8—50.3%. This finding is in line with some reports^12,15^. However, for some tree species, the pantropical model performs well; for example, Chave et al.^34^ and Brown^36^ did not show a significant bias for tree species *C. macrostachyus* and *V. dainellii* respectively. This suggests that the bias of the pantropic generic allometric equation varies between tree species^16^. The tested site-specific allometric equation overestimated the biomass for tree species such as *S. guineense* (71.9%) and *C. macrostachyus* (37.4%) and did not exhibit significant bias towards the other tree species. The performance of the site-specific and pantropic generic model in the forest biomass estimation leads to some uncertainties^46^. Species-specific allometric equation plays an important role in reducing the uncertainty associated with the estimation of biomass. Whenever there is a lack of species-specific allometric equation site-specific model is more important^47^ for the estimation of above-ground biomass than a pantropical model. Tree allometry is affected by the environmental conditions and nature of the tree, while the pantropic model data was collected from outside the Ethiopia; Brown^36^, collected from Central and South America and Southeast Asia; Chave et al.^34^ collected Tropical America and Asia; but Chave et al.^33^ incorporated some tree species as part of Africa. Due to the above-mentioned factor, the application of the pantropical model leads to uncertainty, and using a species-specific allometric model reduces the uncertainty in biomass estimation.

## Conclusions

Interest in the estimation of the biomass and carbon sequestration potential of the forest increased because of the result-based incentive in forest management and conservation. In this regard, allometric equations give an insight into the potential of an intervention, and how much biomass and carbon are stored in the forest. Species-specific allometric equations for the estimation of above-ground biomass were developed for five tree species. The developed models have a great role in improving the accuracy of biomass estimation. Models that used only dbh will have importance in reducing cost in the measurement. The best-ranked allometric equation compared with the allometric equation developed as a site-specific and pantropic generic equation; however, the developed species-specific allometric equation showed better accuracy in the estimation of aboveground tree biomass. The developed biomass model was applied to a Moist evergreen Afromontane Forest, considering the diameter range for each species. However, to improve the model further, it is necessary to include sample trees from various locations within the forest ecosystem.

### Supplementary Information

Supplementary Information 1.

Supplementary Information 2.

## Data Availability

The datasets used and/or analyzed during the current study are available from the corresponding author upon reasonable request.
