# Supplementary material for: Species-specific allometric models for reducing uncertainty in estimating above ground biomass at Moist Evergreen Afromontane Forest of Ethiopia
Source: Sci Rep. 2024 Jan 11;14:1147. doi: 10.1038/s41598-023-51002-6 (PMC10784490; doi:10.1038/s41598-023-51002-6)
Supplement: Supplementary file 1 — Supplementary Information 1. [file 41598_2023_51002_MOESM1_ESM.docx]

**Appendix 1:** Tested above-ground biomass allometric equation for all tree species.

| **Species** | **Model** | **Parameter** | | | | **R^^2^** | **RMSE** | | **MPE** | | **AIC** |
| --- | --- | --- | --- | --- | --- | --- | --- | --- | --- | --- | --- |
|  |  | **a** | **b** | **c** | **d** |  | **kg** | **%** | **kg** | **%** |  |
| *A. gummifera* | M1 | 0.1452** | 2.441*** |  |  | 0.989 | 384.19 | 14.20 | -26.4 | -0.98 | 161 |
|  | M2 | 0.197 * | 2.577*** | -0.266 |  | 0.996 | 240.24 | 8.88 | -12.0 | -0.44 | 161 |
|  | M3 | 0.086* | 2.49*** |  |  | 0.978 | 547.76 | 20.25 | 50.3 | 1.86 | 169 |
|  | M4 | 0.141** | 2.343*** | 0.151 |  | 0.987 | 411.22 | 15.20 | 9.7 | 0.36 | 162 |
|  | M5 | 0.110 | 2.634*** | -0.263 |  | 0.965 | 682.96 | 25.25 | 94.8 | 3.51 | 170 |
|  | M6 | 0.193* | 2.482*** | -0.275 | 0.154 | 0.994 | 292.79 | 10.82 | 33.6 | 1.24 | 162 |
| *C. macrostachyus* | M1 | 0.085* | 2.487*** |  |  | 0.924 | 392.90 | 33.44 | 43.7 | 3.72 | 170 |
|  | M2 | 0.106 | 2.522*** | -0.112 |  | 0.918 | 407.91 | 34.72 | 44.5 | 3.79 | 172 |
|  | M3 | 0.085* | 2.392*** |  |  | 0.970 | 245.29 | 20.88 | 31.2 | 2.65 | 173 |
|  | M4 | 0.083 | 2.503*** | -0.014 |  | 0.919 | 406.89 | 34.64 | 43.0 | 3.66 | 172 |
|  | M5 | 0.056 | 2.314*** | 0.230 |  | 0.948 | 324.98 | 27.66 | 48.2 | 31.21 | 174 |
|  | M6 | 0.105 | 2.525*** | -0.112 | -0.003 | 0.948 | 324.98 | 27.66 | 48.6 | 4.13 | 174 |
| *S. guineense* | M1 | 0.288*** | 2.177*** |  |  | 0.993 | 158.13 | 10.20 | -62.7 | -4.04 | 126 |
|  | M2 | 0.079 | 1.836*** | 0.886* |  | 0.996 | 118.34 | 7.63 | -31.8 | -2.05 | 127 |
|  | M3 | 0.132 | 2.25*** |  |  | 0.958 | 381.45 | 24.60 | 158.4 | 10.22 | 150 |
|  | M4 | 0.22*** | 2.533*** | 0.425* |  | 0.999 | 60.91 | 3.93 | 12.1 | 0.78 | 128 |
|  | M5 | 0.008 | 1.473*** | 2.027** |  | 0.992 | 167.79 | 10.82 | 33.1 | 2.14 | 134 |
|  | M6 | 0.113 | 2.157*** | 0.540 | -0.223 | 0.999 | 60.94 | 3.93 | -7.6 | -0.49 | 128 |
| *V. dainellii* | M1 | 0.099** | 2.652*** |  |  | 0.927 | 198.52 | 30.60 | 52.4 | 8.08 | 110 |
|  | M2 | 0.182 | 4.114 | 2.067* |  | 0.992 | 67.68 | 10.43 | 26.5 | 4.08 | 102 |
|  | M3 | 0.073** | 2.609 *** |  |  | 0.720 | 389.02 | 59.97 | -0.7 | -0.11 | 120 |
|  | M4 | 0.096* | 2.735*** | 0.085 |  | 0.919 | 209.41 | 32.28 | 56.8 | 8.76 | 112 |
|  | M5 | 0.061 | 2.065* | 0.789 |  | 0.918 | 211.22 | 32.56 | 49.6 | 7.64 | 121 |
|  | M6 | 0.183* | 4.109*** | 2.071* | 0.008 | 0.986 | 85.45 | 13.17 | 31.3 | 4.83 | 104 |
| *B. abyssinica* | M1 | 0.255* | 2.249*** |  |  | 0.893 | 114.94 | 29.57 | -17.0 | -4.38 | 98 |
|  | M2 | 0.194 | 2.117*** | 0.270 |  | 0.892 | 115.03 | 29.60 | -21.5 | -5.53 | 104 |
|  | M3 | 0.200 | 2.235*** |  |  | 0.825 | 146.57 | 37.71 | -18.1 | -4.65 | 108 |
|  | M4 | 0.224* | 2.178*** | 0.177 |  | 0.939 | 86.62 | 22.29 | -18.2 | -4.68 | 100 |
|  | M5 | 0.141 | 2.044*** | 0.387 |  | 0.826 | 146.20 | 37.62 | -27.2 | -7.00 | 109 |
|  | M6 | 0.185 | 2.079*** | 0.201 | 0.174 | 0.929 | 93.17 | 23.97 | -30.3 | -7.79 | 101 |
