# Supplementary material for: Species-specific allometric models for reducing uncertainty in estimating above ground biomass at Moist Evergreen Afromontane Forest of Ethiopia
Source: Sci Rep. 2024 Jan 11;14:1147. doi: 10.1038/s41598-023-51002-6 (PMC10784490; doi:10.1038/s41598-023-51002-6)
Supplement: Supplementary file 2 — Supplementary Information 2. [file 41598_2023_51002_MOESM2_ESM.docx]

**Appendix 2:** Tested biomass allometric equation for tree compartment in all tree species.

| **Species** | **Tree compartment** | **model** | **parameter** | | | | **R^2** | **RMSE** | | **MPE** | | **AIC** |
| --- | --- | --- | --- | --- | --- | --- | --- | --- | --- | --- | --- | --- |
|  |  |  | **A** | **b** | **c** | **d** |  | **kg** | **%** | **kg** | **%** |  |
| *A. gummifera* | Foliage | M1 | 0.119 | 1.827*** |  |  | 0.814 | 66.0 | 42.31 | -9.3 | -5.95 | 122 |
|  |  | M2 | 0.069 | 1.552** | 0.513 |  | 0.560 | 101.5 | 65.10 | -9.6 | -6.16 | 123 |
|  |  | M3 | 0.079* | 1.867** |  |  | 0.860 | 57.3 | 36.73 | -8.6 | -5.53 | 118 |
|  |  | M4 | 0.111 | 1.486** | 0.514 |  | 0.865 | 56.2 | 36.06 | -7.9 | -5.09 | 122 |
|  |  | M5 | 0.058 | 1.685*** | 0.329 |  | 0.715 | 81.7 | 52.39 | -8.0 | -5.16 | 119 |
|  |  | M6 | 0.061 | 1.177* | 0.534 | 0.553 | 0.631 | 92.9 | 59.58 | -4.8 | -3.06 | 123 |
|  | Branch | M1 | 0.008 | 2.909*** |  |  | 0.988 | 194.0 | 17.19 | 1.1 | 0.09 | 145 |
|  |  | M2 | 0.013* | 3.208*** | 0.541* |  | 0.933 | 454.1 | 40.23 | -14.5 | -1.28 | 141 |
|  |  | M3 | 0.002 | 3.14*** |  |  | 0.974 | 283.9 | 25.15 | 35.8 | 3.17 | 155 |
|  |  | M4 | 0.006 | 2.761*** | 0.289 |  | 0.983 | 230.1 | 20.38 | 35.9 | 3.18 | 146 |
|  |  | M5 | 0.003 | 3.695*** | 0.814** |  | 0.979 | 253.0 | 22.41 | 47.2 | 4.18 | 155 |
|  |  | M6 | 0.01* | 3.05*** | 0.585** | 0.381 | 0.930 | 463.8 | 41.09 | 25.1 | 2.22 | 141 |
|  | M. stem | M1 | 0.151** | 2.285*** |  |  | 0.977 | 273.1 | 19.23 | -49.4 | -3.48 | 158 |
|  |  | M2 | 0.134 | 2.23*** | 0.113 |  | 0.949 | 401.7 | 28.28 | -41.3 | -2.90 | 159 |
|  |  | M3 | 0.099* | 2.316*** |  |  | 0.943 | 428.1 | 30.14 | -130.6 | -9.19 | 159 |
|  |  | M4 | 0.15* | 2.222*** | 0.094 |  | 0.943 | 428.1 | 30.14 | -130.6 | -9.19 | 159 |
|  |  | M5 | 0.089 | 2.248*** | 0.121 |  | 0.925 | 489.4 | 34.46 | 6.6 | 0.47 | 161 |
|  |  | M6 | 0.135 | 2.17*** | 0.103 | 0.089 | 0.946 | 413.4 | 29.10 | -28.8 | -2.03 | 161 |
| *C. macrostachyus* | Foliage | M1 | 0.016 | 2.183*** |  |  | 0.899 | 22.1 | 33.90 | -0.8 | -1.16 | 117 |
|  |  | M2 | 0.025 | 2.24*** | 0.213 |  | 0.883 | 23.7 | 36.45 | -1.6 | -2.48 | 119 |
|  |  | M3 | 0.016 | 2.093*** |  |  | 0.877 | 24.4 | 37.47 | -0.9 | -1.34 | 119 |
|  |  | M4 | 0.133 | 0.622 | 1.456* |  | 0.899 | 22.1 | 33.89 | 0.7 | 1.04 | 111 |
|  |  | M5 | 0.014 | 2.067*** | 0.090 |  | 0.867 | 25.3 | 38.91 | -1.5 | -2.31 | 121 |
|  |  | M6 | 0.397 | 0.757 | 0.573 | 1.529 | 0.897 | 22.3 | 34.27 | 0.1 | 0.21 | 113 |
|  | Branch | M1 | 0.002 | 3.047*** |  |  | 0.947 | 103.8 | 31.15 | -6.9 | -2.07 | 154 |
|  |  | M2 | 0.113 | 3.546*** | 1.807* |  | 0.922 | 126.4 | 37.90 | -15.3 | -4.60 | 150 |
|  |  | M3 | 0.003 | 2.851*** |  |  | 0.910 | 135.8 | 40.73 | -15.4 | -4.63 | 155 |
|  |  | M4 | 0.001 | 3.774*** | 0.650 |  | 0.827 | 187.9 | 56.35 | -32.7 | -9.80 | 154 |
|  |  | M5 | 0.056 | 3.246*** | 1.375 |  | 0.772 | 216.1 | 64.83 | -36.6 | -10.97 | 154 |
|  |  | M6 | 0.071 | 3.721*** | 1.705 | 0.196 | 0.902 | 141.8 | 42.52 | -21.2 | -6.35 | 152 |
|  | M. stem | M1 | 0.11 | 2.32*** |  |  | 0.875 | 326.2 | 42.02 | 23.1 | 2.98 | 165 |
|  |  | M2 | 0.035 | 2.064*** | 0.672 |  | 0.812 | 399.9 | 51.52 | 38.5 | 4.96 | 164 |
|  |  | M3 | 0.093 | 2.273*** |  |  | 0.939 | 227.3 | 29.28 | 14.1 | 1.82 | 171 |
|  |  | M4 | 0.108 | 2.338*** | 0.017 |  | 0.871 | 330.8 | 42.61 | 20.5 | 2.64 | 167 |
|  |  | M5 | 0.017 | 1.853*** | 1.061* |  | 0.829 | 381.7 | 49.17 | 46.3 | 5.97 | 166 |
|  |  | M6 | 0.036 | 2.042** | 0.675 | 0.019 | 0.797 | 415.3 | 53.50 | 37.6 | 4.85 | 166 |
| *S. guineense* | Foliage | M1 | 0.202* | 1.623*** |  |  | 0.888 | 42.5 | 35.50 | 13.7 | 11.39 | 102 |
|  |  | M2 | 0.681 | 1.965** | 0.862 |  | 0.880 | 44.0 | 36.73 | 11.8 | 9.83 | 103 |
|  |  | M3 | 0.054** | 1.872*** |  |  | 0.911 | 37.9 | 31.62 | 7.7 | 6.45 | 91 |
|  |  | M4 | 0.206* | 1.576** | 0.063 |  | 0.882 | 43.7 | 36.46 | 14.4 | 12.00 | 104 |
|  |  | M5 | 0.033 | 1.723*** | 0.371 |  | 0.926 | 34.5 | 28.79 | 3.7 | 3.12 | 93 |
|  |  | M6 | 1.071 | 2.526* | 1.296 | 0.531 | 0.751 | 63.4 | 52.89 | 24.1 | 20.10 | 104 |
|  | Branch | M1 | 0.024*** | 2.533*** |  |  | 0.986 | 101.0 | 15.85 | 17.6 | 2.76 | 96 |
|  |  | M2 | 0.028* | 2.567*** | 0.088 |  | 0.986 | 102.0 | 16.00 | 17.4 | 2.72 | 98 |
|  |  | M3 | 0.008 | 2.657*** |  |  | 0.903 | 269.7 | 42.33 | 114.4 | 17.96 | 121 |
|  |  | M4 | 0.024*** | 2.527*** | 0.008 |  | 0.986 | 102.2 | 16.04 | 17.9 | 2.81 | 98 |
|  |  | M5 | 0.001 | 2.026*** | 1.611* |  | 0.967 | 156.7 | 24.58 | 53.7 | 8.43 | 112 |
|  |  | M6 | 0.029 | 2.621*** | 0.137 | 0.043 | 0.984 | 109.3 | 17.16 | 23.3 | 3.65 | 100 |
|  | M. stem | M1 | 0.237* | 2.075*** |  |  | 0.941 | 213.1 | 26.86 | -76.2 | -9.60 | 165 |
|  |  | M2 | 0.017 | 1.41*** | 1.76** |  | 0.937 | 220.3 | 27.76 | -51.0 | -6.43 | 122 |
|  |  | M3 | 0.136 | 2.097*** |  |  | 0.972 | 148.2 | 18.68 | 34.3 | 4.32 | 143 |
|  |  | M4 | 0.137* | 2.718*** | 0.741* |  | 0.973 | 145.6 | 18.35 | -31.3 | -3.94 | 125 |
|  |  | M5 | 0.004 | 1.15*** | 2.486** |  | 0.953 | 191.3 | 24.11 | -23.4 | -2.95 | 129 |
|  |  | M6 | 0.032 | 1.89** | 1.185 | 0.300 | 0.949 | 198.8 | 25.05 | -48.6 | -6.12 | 123 |
| *V. dainellii* | Foliage | M1 | 0.02 | 2.502*** |  |  | 0.835 | 29.2 | 43.76 | 4.3 | 6.50 | 82 |
|  |  | M2 | 0.143 | 4.402*** | 3.139*** |  | 0.977 | 10.9 | 16.38 | 1.5 | 2.32 | 68 |
|  |  | M3 | 0.088 | 1.945*** |  |  | 0.901 | 22.7 | 33.98 | -0.7 | -1.01 | 85 |
|  |  | M4 | 0.040 | 2.999*** | 0.858 |  | 0.860 | 26.9 | 40.27 | 2.7 | 4.05 | 82 |
|  |  | M5 | 0.112*** | 2.22** | 0.466 |  | 0.848 | 29.0 | 40.92 | -1.9 | -2.66 | 87 |
|  |  | M6 | 0.187* | 4.628*** | 3.108*** | 0.406 | 0.986 | 8.5 | 12.74 | 1.4 | 2.15 | 66 |
|  | Branch | M1 | 0.012* | 3.09*** |  |  | 0.899 | 143.4 | 43.84 | 19.2 | 5.87 | 103 |
|  |  | M2 | 0.017 | 4.086** | 1.366 |  | 0.942 | 108.8 | 33.28 | 9.6 | 2.94 | 104 |
|  |  | M3 | 0.006** | 3.13*** |  |  | 0.561 | 298.5 | 91.26 | -55.3 | -16.90 | 100 |
|  |  | M4 | 0.011 | 3.251*** | 0.147 |  | 0.903 | 140.0 | 42.82 | 11.3 | 3.45 | 105 |
|  |  | M5 | 0.006* | 3.249** | 0.166 |  | 0.729 | 76.8 | 40.90 | 9.6 | 5.11 | 101 |
|  |  | M6 | 0.015 | 4.088* | 1.252 | 0.077 | 0.938 | 112.2 | 34.31 | 6.2 | 1.89 | 106 |
|  | M. stem | M1 | 0.062** | 2.504*** |  |  | 0.851 | 81.4 | 39.20 | -6.3 | -3.04 | 77 |
|  |  | M2 | 0.054* | 2.218** | 0.409 |  | 0.750 | 105.6 | 50.85 | -10.2 | -4.90 | 79 |
|  |  | M3 | 0.062 | 2.389*** |  |  | 0.832 | 74.1 | 39.46 | -15.2 | 8.39 | 97 |
|  |  | M4 | 0.062* | 2.488*** | 0.020 |  | 0.878 | 73.8 | 35.51 | -0.6 | -0.28 | 79 |
|  |  | M5 | 0.029 | 1.065 | 2.034 |  | 0.878 | 73.7 | 35.46 | 19.1 | 9.21 | 93 |
|  |  | M6 | 0.054 | 2.22** | 0.447 | 0.035 | 0.820 | 89.5 | 43.09 | -5.6 | -2.69 | 81 |
| *B. abyssinica* | Foliage | M1 | 0.148 | 1.63*** |  |  | 0.674 | 11.8 | 43.05 | -1.5 | -5.62 | 73 |
|  |  | M2 | 0.455 | 2.178* | 1.126 |  | 0.592 | 13.3 | 48.20 | -1.4 | -4.98 | 73 |
|  |  | M3 | 0.152 | 1.563*** |  |  | 0.644 | 12.4 | 44.98 | -1.0 | -3.73 | 76 |
|  |  | M4 | 0.098 | 1.506** | 0.398 |  | 0.808 | 9.1 | 33.02 | -0.8 | -2.99 | 72 |
|  |  | M5 | 0.275 | 1.878* | 0.644 |  | 0.056 | 20.2 | 73.27 | -4.1 | -14.95 | 78 |
|  |  | M6 | 0.245 | 2.092** | 1.113 | 0.418 | 0.803 | 9.2 | 33.48 | -1.8 | -6.68 | 70 |
|  | Branch | M1 | 0.061 | 2.395*** |  |  | 0.756 | 74.8 | 48.57 | -11.0 | -7.17 | 98 |
|  |  | M2 | 0.109 | 2.656*** | 0.552 |  | 0.468 | 110.3 | 71.64 | -24.5 | -15.89 | 99 |
|  |  | M3 | 0.047 | 2.381*** |  |  | 0.721 | 80.0 | 51.93 | -9.6 | -6.21 | 99 |
|  |  | M4 | 0.050 | 2.352*** | 0.166 |  | 0.768 | 72.9 | 47.33 | -14.0 | -9.11 | 99 |
|  |  | M5 | 0.072 | 2.592** | 0.442 |  | 0.293 | 127.3 | 82.63 | -27.1 | -17.61 | 101 |
|  |  | M6 | 0.086 | 2.608** | 0.535 | 0.166 | 0.239 | 132.0 | 85.72 | -43.1 | -27.98 | 100 |
|  | M. stem | M1 | 0.130 | 2.262*** |  |  | 0.920 | 51.8 | 25.01 | -7.4 | -3.58 | 96 |
|  |  | M2 | 0.045* | 1.674*** | 1.145** |  | 0.986 | 21.7 | 10.49 | -0.6 | -0.29 | 85 |
|  |  | M3 | 0.091 | 2.283*** |  |  | 0.825 | 76.8 | 37.10 | -10.7 | -5.19 | 100 |
|  |  | M4 | 0.130 | 2.171*** | 0.148 |  | 0.948 | 42.0 | 20.26 | -8.3 | -3.99 | 97 |
|  |  | M5 | 0.033 | 1.636*** | 1.234** |  | 0.973 | 30.4 | 14.66 | -3.6 | -1.75 | 90 |
|  |  | M6 | 0.051* | 1.638*** | 1.046** | 0.125 | 0.987 | 20.9 | 10.11 | 0.7 | 0.33 | 83 |
